# Ischemia-reperfusion injury in a rat microvascular skin free flap model: A histological, genetic, and blood flow study

**DOI:** 10.1371/journal.pone.0209624

**Published:** 2018-12-27

**Authors:** Alberto Ballestín, Javier G. Casado, Elena Abellán, F. Javier Vela, Verónica Álvarez, Alejandra Usón, Esther López, Federica Marinaro, Rebeca Blázquez, Francisco Miguel Sánchez-Margallo

**Affiliations:** 1 Department of Microsurgery, Jesús Usón Minimally Invasive Surgery Centre, Cáceres, Spain; 2 Stem Cell Therapy Unit, Jesús Usón Minimally Invasive Surgery Centre, Cáceres, Spain; Medical University Graz, AUSTRIA

## Abstract

Ischemia reperfusion injury is associated with tissue damage and inflammation, and is one of the main factors causing flap failure in reconstructive microsurgery. Although ischemia-reperfusion (I/R) injury is a well-studied aspect of flap survival, its biological mechanisms remain to be elucidated. To better understand the biological processes of ischemia reperfusion injury, and to develop further therapeutic strategies, the main objective of this study was to identify the gene expression pattern and histological changes in an I/R injury animal model. Fourteen rats (n = 7/group) were randomly divided into control or ischemia-reperfusion group (8 hours of ischemia). Microsurgical anastomoses were objectively assessed using transit-time-ultrasound technology. Seven days after surgery, flap survival was evaluated and tissue samples were harvested for anatomopathological and gene-expression analyses.The I/R injury reduced the survival of free flaps and histological analyses revealed a subcutaneous edema together with an inflammatory infiltrate. Interestingly, the Arginase 1 expression level as well as the ratio of Arginase 1/Nitric oxide synthase 2 showed a significant increase in the I/R group. In summary, here we describe a well-characterized I/R animal model that may serve to evaluate therapeutic agents under reproducible and controlled conditions. Moreover, this model could be especially useful for the evaluation of arginase inhibitors and different compounds of potential interest in reconstructive microsurgery.

## Introduction

Microsurgical free-tissue transfer has become a common practice in reconstructive surgery to provide an efficient approach to restore the form and function after complex tissue defects due to trauma injuries or after oncological resections. The loss of skin, muscle and even bone especially after avulsion or amputation injuries or after tumor resections in the head, neck, trunk or extremities, represent reconstructive challenges for surgeons. Microsurgical reconstructions with composite or single free-tissue transfers are ideal options to cover these three-dimensional defects [1,2]. Well-perfused tissues are required to fill the defect, providing functional and aesthetic outcomes.

Although literature from previous studies shows that the final success rate of microsurgical free flap transfer is 90–95% [3,4,5,6,7,8,9], up to 25% of transferred flaps need revision because of complications [10,11,12,13]. These postoperative complications can be due to surgical problems (i.e., technical errors, prolonged tissue ischemia or venous congestion) [14,15], or the pathological condition of the patient (i.e., diabetes, hypertension or radiated patient) [16,17,18].

One of the most studied biological processes affecting flap survival is ischemia-reperfusion injury [3,4,19], which may lead to complete flap failure. In fact, it is important to note that, even partial flap necrosis leads to suboptimal aesthetic and functional results [20].

During last two decades, several research studies attempted attenuation of ischemia-reperfusion injury using different compounds and molecules [21,22,23,24,25,26]. Most of the studies have shown positive or promising results in preclinical settings [21,22,27,28] and some clinical trials are being conducted in this field (clinicaltrials.gov identifiers NCT01905501 and NCT03535623).

Any free flap transfer includes a period of ischemia followed by reperfusion. The ischemic interval lasts from completion of flap harvesting till the end of the microvascular anastomoses. When the ischemic period exceeds the tolerance of the tissue, an apoptotic/necrotic process is initiated. Additionally, a wide range of pathophysiological events occurs within the tissue after reperfusion [6,19,23]. Reactive oxygen species have been shown to trigger reperfusion injury and lead to recruitment of proinflammatory neutrophils in ischemic tissues [20].

The incidence of complications after flap transfer necessitates better understanding of the pathophysiological processes of ischemia reperfusion injury, which are associated with different biological/physiological processes such as metabolic dysfunction, cell death, hypoxia, inflammation and apoptosis or necrosis [29]. Gene expression analysis of a translational I/R model is crucial for evaluation of these biological processes and potential targets for therapeutic interventions.

Animal models are of major importance in understanding the pathophysiology of various biological processes in humans at the cellular and molecular levels [30,31]. Appropriate animal models are essential to predict the value and effect of novel therapeutic approaches and to assess the efficacy of new drugs [32]. With this background, the main objective of this study was to describe a clinically relevant model appropriate to conduct preclinical studies in the field of ischemia-reperfusion injury in reconstructive microsurgery.

Aiming at this, we firstly optimized the microsurgical and anatomical approach to harvest the free flap model. Secondly, the patency of the microvascular anastomoses was assessed using transit-time ultrasound technology. Third, post-mortem analyses were carried out to evaluate the histological changes. Finally the gene expression analysis was performed for a set of genes involved in inflammation, oxidative stress, angiogenesis and apoptosis.

The analysis of inflammatory-related genes was focused on Th1/Th2 cytokines (Interleukin 1 beta, Interleukin 6, Interleukin 10, Tumor Necrosis Factor) as well as on M1/M2-related mRNA expression (Arginase 1, Nitric Oxide Synthase 2), which has been found to be closely involved with ischemia reperfusion [33] and ischemic heart failure [34]. In the case of angiogenesis, this analysis was limited to the quantification of angiopoietin-2 (widely described in myocardial infarction and cerebral ischemia) [35,36], Fibroblast growth factor 2 and Vascular endothelial growth factor A (both related to hindlimb ischemia models) [37,38]. For oxidative-stress related genes, the analysis was focused on Superoxide dismutase 1 and Hypoxia-inducible factor 1, alpha subunit. In the case of Hypoxia-inducible factor 1, alpha, it has been shown to be differentially expressed in the context of microsurgical free muscle tissue transfer [39,40] and Superoxide dismutase 1 has been evaluated in an animal model for flap survival evaluation [41]. Finally, the analysis of apoptosis-related genes included the analysis of Caspase 3, Caspase 9, B-cell CLL/lymphoma 2. These proteins have a key role in the regulation of apoptosis [42,43], being frequently used to evaluate ischemia induction in animal models [44,45,46].

In summary, here we describe a well-characterized I/R animal model for the evaluation of therapeutic agents under reproducible and controlled conditions. This model could be especially interesting in reconstructive microsurgery and preclinical studies.

## Materials and methods

### Experimental design

Adult male Wistar rats (n = 14), weighing 290–350 g, were used in this study. The experimental procedures were approved by the ethical committee of animal experimentation of the Jesús Usón Minimally Invasive Surgery Center and were in accordance with the welfare standards of the regional government which are based on European legislation.

Previous to surgery, the rats were acclimatized in cages at 22–25°C with food and water ad libitum. All surgical procedures were performed under general inhalation anesthesia using sevoflurane. Enrofloxacin (7.5 mg/kg/day during 5 days), a broad spectrum antibiotic, and Meloxicam (1 mg/kg/day during 5 days), an anti-inflammatory and analgesic drug, were injected subcutaneously in all the animals after completion of the procedures. All animals were individually housed after surgery and postoperative protectors (patent number P201400272, Spanish Patent and Trademark Office) were placed on them to impede self-mutilation and injuries.

We used a caudal superficial epigastric skin free flap (3 × 6 cm) in our study. The surgical procedures were performed in 14 animals, randomly divided into two groups. The raised flaps in the animals of the control group (*n* = 7) did not suffer any ischemic insult (the vascular pedicle was not cut). The raised flaps in the ischemia-reperfusion group (*n* = 7) were subjected to ischemia for 8 hours prior to revascularization (detailed in the flap model section).

Blood flow of the flap pedicle was measured using a transit-time ultrasound flowmeter and microvascular probes (Transonic Systems Inc., Ithaca, NY) prior to repositioning of the skin to its original site using 4/0 silk sutures following a simple interrupted pattern. Microsurgical arterial and venous anastomoses were performed using 10/0 nylon sutures in the animals of the ischemia-reperfusion group (I/R Group). After the surgery, pictures were taken for planimetric analysis.

**Fig 1:**
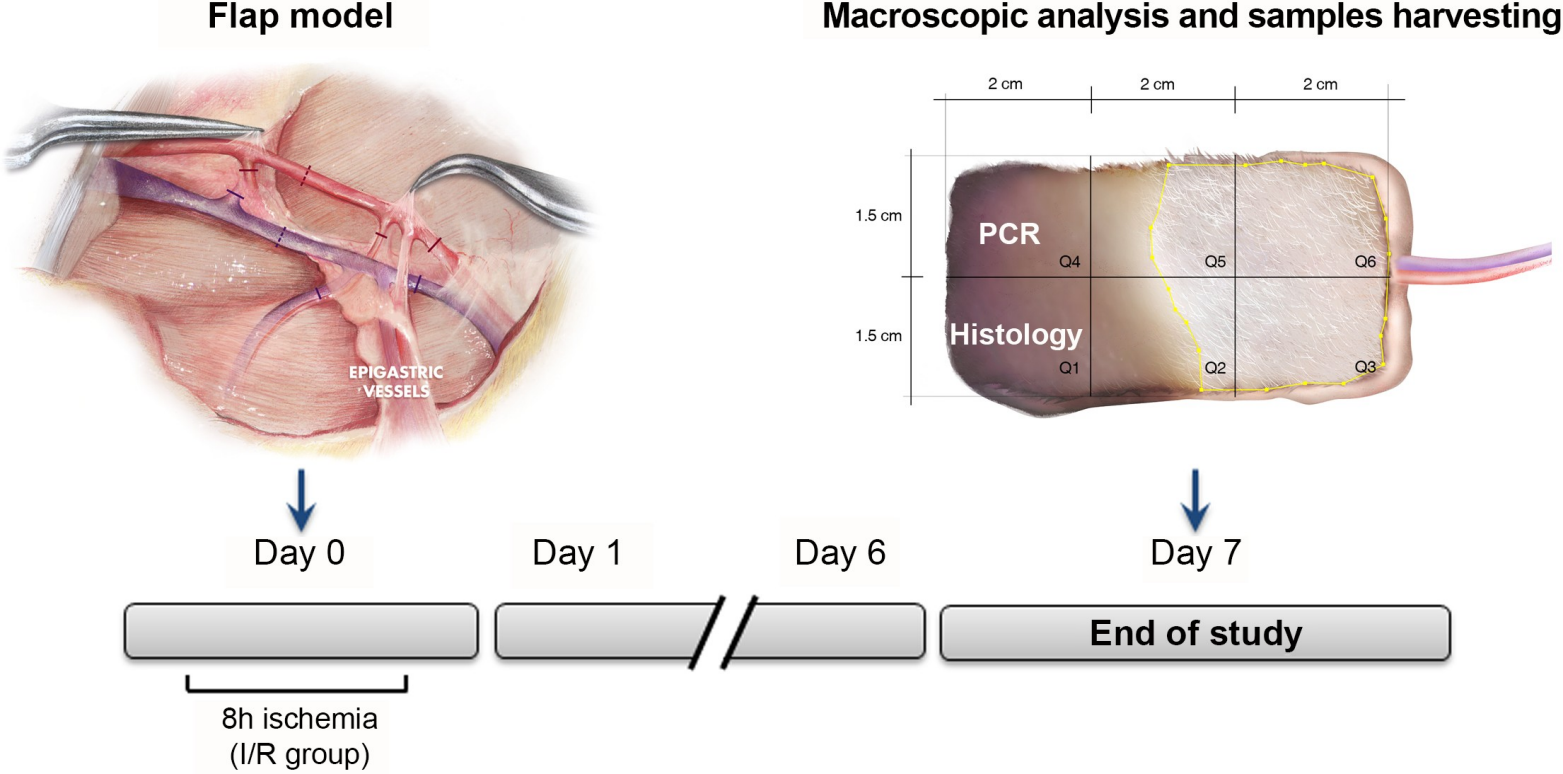
Experimental design.

Seven days post-surgery the rats were anesthetized again. Macroscopic pictures were taken to assess the survival area of the flaps, then the flaps were raised again, the blood flow was evaluated, and the flaps were divided for histopathological and quantitative reverse transcription polymerase chain reaction (RT-qPCR) analysis. Then, the animals were euthanized by rapid intracardiac injection of KCl (2 meq/kg, KCl 2M), under general inhalation anesthesia, according to the ethical committee recommendations. A schematic representation of the experimental design is shown in Fig 1.

### Flap model

**Fig 2:**
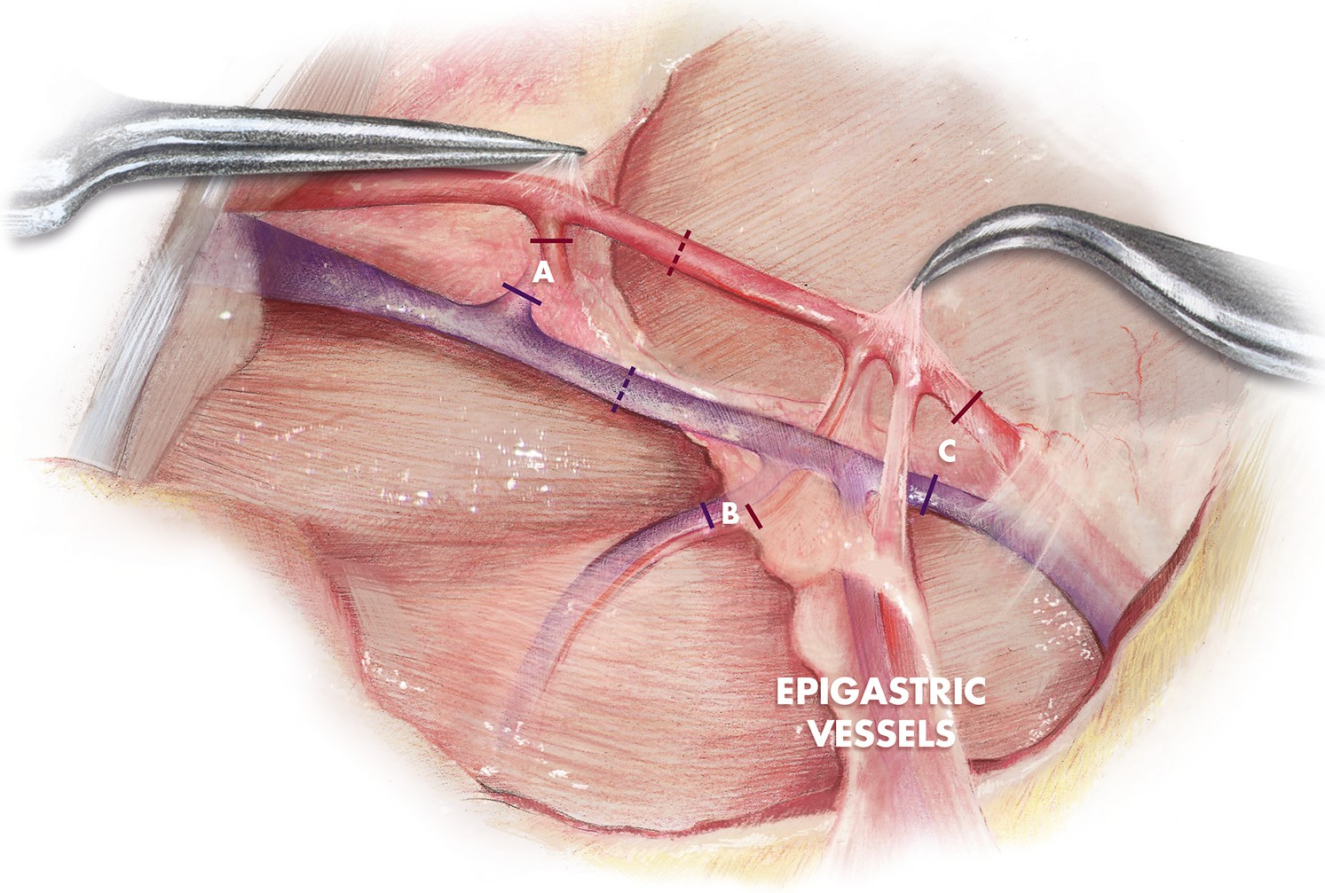
Flap model. A) Ligatures of lateral circumflex femoral artery and vein. B) Ligatures of proximal caudal femoral artery and vein. C) Ligatures of saphenous artery and vein. Dotted lines represent the microsurgical anastomoses sites.

The experimental study was based on a free inguinal cutaneous flap [47]. A 3 x 6-cm skin flap was raised, being perfused by the left superficial caudal epigastric vessels. After dissection of the vascular pedicle, ligatures with 8/0 nylon sutures were performed on the proximal caudal femoral vessels, on the lateral circumflex femoral vessels and on the saphenous artery and vein [48] (Fig 2).

After completing the dissection, the flaps of the control group were relocated free of ischemia in their original site. The ischemia was induced in I/R group by cutting the artery and vein of the axial pattern flaps. Heparinized saline solution was used to perfuse the flap and thus remove stagnant blood from the microcirculation. Then, microsurgical anastomoses were performed (10/0 nylon suture) and removal of the clamps was done after the 8h of ischemia period.

### Vascular patency assessment using transit-time ultrasound technology

Transit-time ultrasound flowmeter and microvascular probes (Transonic Systems Inc., Ithaca, N.Y.) were used to verify blood flow patency intraoperatively after the end-to-end anastomoses performed in the animals of the I/R group and also to verify again the blood flow one week after the procedure. Blood flow was also measured in the control group the day of the surgery and at the end of the study.

### Macroscopic measurement of the flap survival

A macroscopic follow-up of the flaps was carried out evaluating the changes. Seven days after surgery, the surviving flap areas were measured by image analysis using the ImageJ software (program designed for scientific multidimensional images). The areas of survival and necrosis were measured in cm^2^ and the percentage of viable area was calculated as: (cm^2^ of viable area/ cm^2^ of total flap area) x 100.

### Sample harvesting

**Fig 3:**
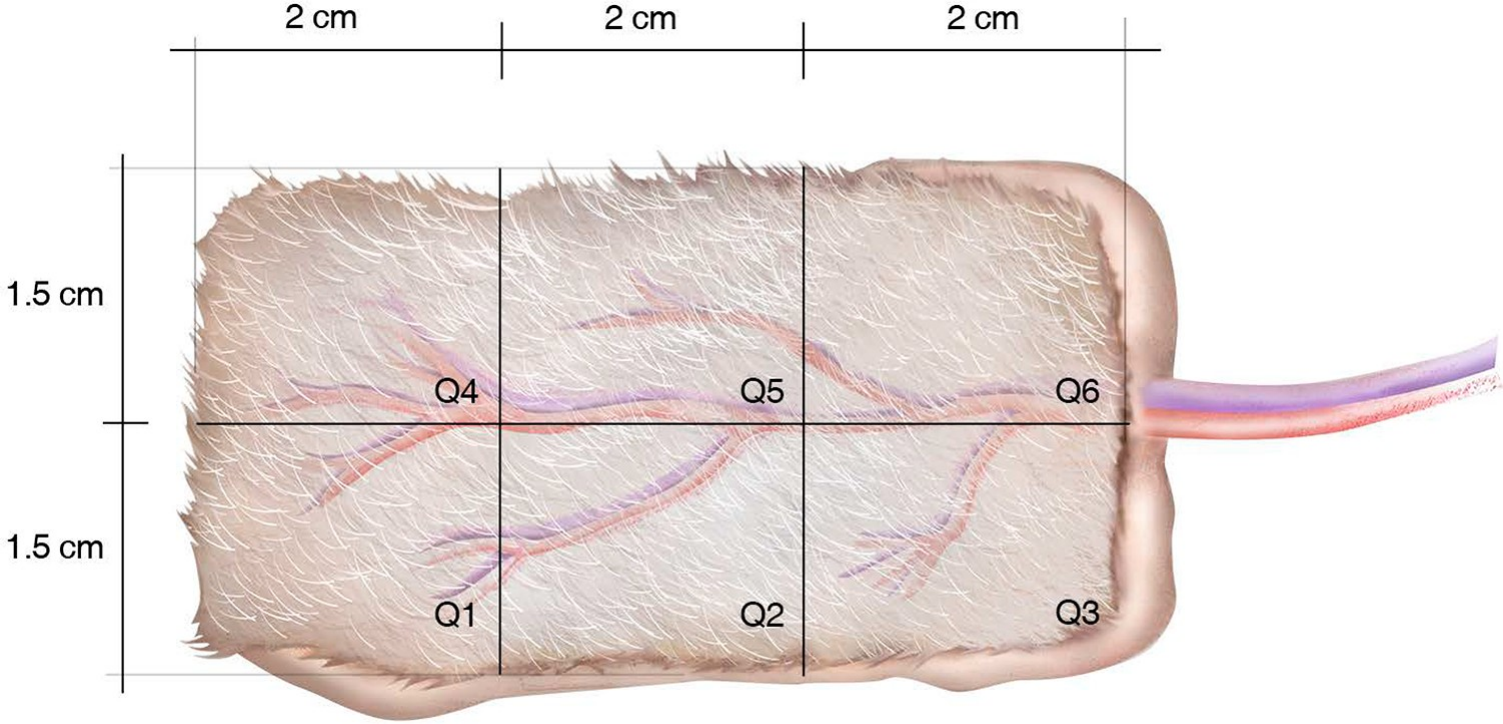
Tissue sampling diagram. The quadrants Q1 were excised and fixed in 4% paraformaldehyde for further histological analysis. The quadrants Q4 were cryopreserved at -80°C for gene expression analysis.

On day 7, animals were anesthetized for tissue sample harvesting. All flaps were divided in 6 quadrants of 2 cm x 1.5 cm (Fig 3). Tissue samples from quadrants Q1 were fixed with 4% paraformaldehyde and were paraffin-embedded. Tissues were sliced at 4 μm for histological analysis. Tissue samples from quadrants Q4 were cryopreserved at -80°C till tested for RT-qPCR analysis. Greater tissue damage was found in the distal area of flaps. We focused our analyses on Q1 and Q4, which are the more distal quadrants to the flap vascular pedicle.

### Histological analysis

Paraffin-embedded samples were processed for hematoxylin/eosin staining and for Masson’s Trichrome staining. All preparations were visualized, scored and evaluated under light microscopy. A blinded histopathological evaluation of all samples was done.

The severity of inflammation for each sample was scored according to the presence of neutrophils, eosinophils, lymphocytes, plasma cells, macrophages, mastocytes and giant cells as follows: (1) Mild/without formation of aggregates, (2) Moderate/occasional aggregates, (3) Intense/packed. The severity of tissue damage was scored for necrosis, edema, hemorrhage and thrombosis as follows: (0) absent, (1) mild, (2) moderate, (3) intense. Fibrosis and vascular proliferation were assessed from the Masson’s Trichrome slides and scored as follows: (1) mild, (2) moderate and (3) intense.

### Methodological gene selection

The selection of genes was performed according to Gene Ontology classification [49,50]. The first selection criteria was based on the rat (*Rattus norvegicus*) genes that were involved in the following biological processes with proved experimental evidence: response to ischemia (GO:0002931), regulation of inflammatory response (GO:0050729), regulation of angiogenesis (GO:0045766), regulation of necrotic cell death (GO:0010940), regulation of execution phase of apoptosis (GO:1900117), regulation response to oxidative stress (GO:0036473, GO:1902883, GO:1902884) and cornification (GO:0070268). A total of 165 genes were obtained. Then, we investigated these genes that regulate the same biological processes in humans (with proved experimental evidence), which lead to selection of 55 genes. From those, we selected Arginase, iNOS, TNF-α, IL-1β, IL6 and IL10 for monitoring the inflammatory response. In order to analyze the regulation of angiogenesis, we quantified VEGF, FGF and angiopoietin-2. Finally, HiF, SOD1, Bcl-2, Cas3 and Cas9 were used to assess the necrosis/apoptosis and oxidative stress processes. Gusb and 18S ribosomal RNA were used as housekeeping genes. Gene symbol, gene name, gene ID, Rat Genome Database (RGD) reference and ThemoFisher Assay ID Genes IDs are listed in Table 1.

**Table 1:** List of selected genes belonging to inflammatory response, angiogenesis, necrosis/apoptosis and oxidative stress pathways.

Gene symbol (Gene name)	Gene ID	RGD	ThemoFisher Assay ID
Angpt2 (Angiopoietin 2)	Rn.138360	RGD:621861	Rn01756774_m1
Arg1 (Arginase 1)	Rn.9857	RGD:2150	Rn00691090_m1
Bcl2 (B-cell CLL/lymphoma 2)	Rn.9996	RGD:2199	Rn99999125_m1
Casp3 (Caspase 3)	Rn.10562	RGD:2275	Rn00563902_m1
Casp9 (Caspase 9)	Rn.32199	RGD:61867	Rn00581212_m1
Fgf2 (Fibroblast growth factor 2)	Rn.31808	RGD:2609	Rn00570809_m1
Gusb (Glucuronidase, beta)	Rn.3692	RGD:2772	Rn00566655_m1
Hif1a (Hypoxia-inducible factor 1, alpha subunit)	Rn.10852	RGD:61928	Rn01472831_m1
IL1b (Interleukin 1 beta)	Rn.9869	RGD:2891	Rn00580432_m1
IL6 (Interleukin 6)	Rn.9873	RGD:2901	Rn01410330_m1
IL10 (Interleukin 10)	Rn.9868	RGD:2886	Rn01644839_m1
Nos2 (Nitric oxide synthase 2, inducible)	Rn.10400	RGD:3185	Rn00561646_m1
Rn18s (18S ribosomal RNA)	Rn.1868	RGD:2189	Rn03928990_g1
Sod1 (Superoxide dismutase 1, soluble)	Rn.6059	RGD:3731	Rn00566938_m1
Tnf (Tumor necrosis factor)	Rn.2275	RGD:3876	Rn99999017_m1
Vegfa (Vascular endothelial growth factor A)	Rn.1923	RGD:619991	Rn01511602_m1

### RNA isolation, reverse transcription and real time polymerase chain reaction

Tissues were disrupted and homogenized in 1 ml of TRI-Reagent (Sigma, St. Louis, MO, USA). Then, chloroform was added and samples were incubated for 5–10 min at room temperature. After centrifugation of 15 min at 12,000 *g*, the aqueous phase was mixed with 500 μl of isopropanol and incubated at -80°C for 20 min to precipitate the RNA. Consecutive centrifugations and ethanol washings were performed. Finally, the pellets were resuspended in RNase-free 0.1% DEPC water (Invitrogen).

The cDNA was synthesized using iScript Reverse Transcription Supermix (BioRad, Hercules, CA, USA) from 1 μg of RNA in reverse transcription reaction for 20 min at 46°C, after a priming period of 5 min at 25°C and followed by RT inactivation at 95°C for 1 min. For qPCR amplification, commercial gene expression assay kits were used (Thermo Fisher Scientific Inc., Waltham, MA, USA) according to manufacturer recommendations. The reaction was performed using TaqMan probes in a QuantStudio 3 Real-Time PCR System (Applied Biosystems, Thermo Fisher Scientific Inc.) and the products were quantified by fluorescent method using 2^-ΔCt^ expression with Gusb as housekeeping gene.

### Statistical analysis

Statistical analyses were performed using SPSS 21 software (IBM Corp., Armonk, NY). All data are expressed as the mean ± standard deviations. Shapiro–Wilk test was used to determine normality of each variable. For parametric variables, differences between means of two groups were analyzed by a Student’s t-test and Levene’s test was used to evaluate the homogeneity of variance. For non-parametric variables, Mann-Whitney’s U test was used to determine differences between groups. In all analyses, p-values <0.05 were considered to indicate statistically significant differences between groups.

## Results

### Vascular patency assessment using transit-time ultrasound technology

All 28 microsurgical anastomoses were patent 1 week after the surgery day. The results of blood flow before the closure of the skin on the day of the surgery were 0.69 ± 0.26 mL/min for arteries and 0.54 ± 0.17 mL/min for veins. One week later, the results were as follows: 0.88 ± 0.45 mL/min for arteries and 0.83 ± 0.45 mL/min for veins. No significant differences in blood flow were detected between the groups.

### Macroscopic measurement of the flap survival

**Fig 4:**
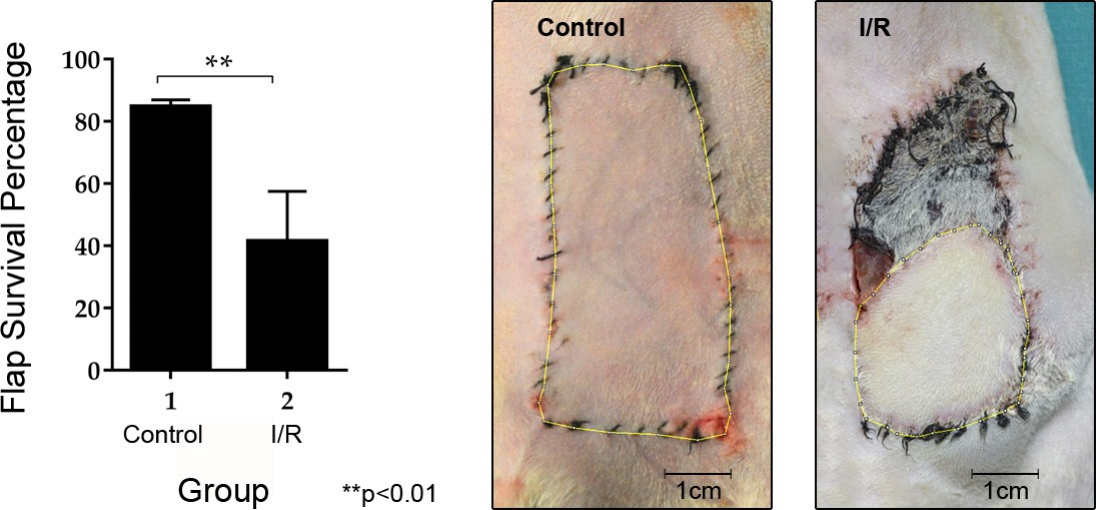
Comparison of skin flap survival areas between groups by Student’s t-test (**p < 0.01) and representative images at 1-week post-surgery.

Flap survival area in the control group was significantly higher than that in flaps that suffered 8 h of ischemic insult (p < 0.01). As shown in Fig 4, the flap survival area in control group was 84.11 ± 2.21%. In contrast, the flap survival area in the I/R group was 41.82 ± 15.99%.

### Histological analysis

**Fig 5:**
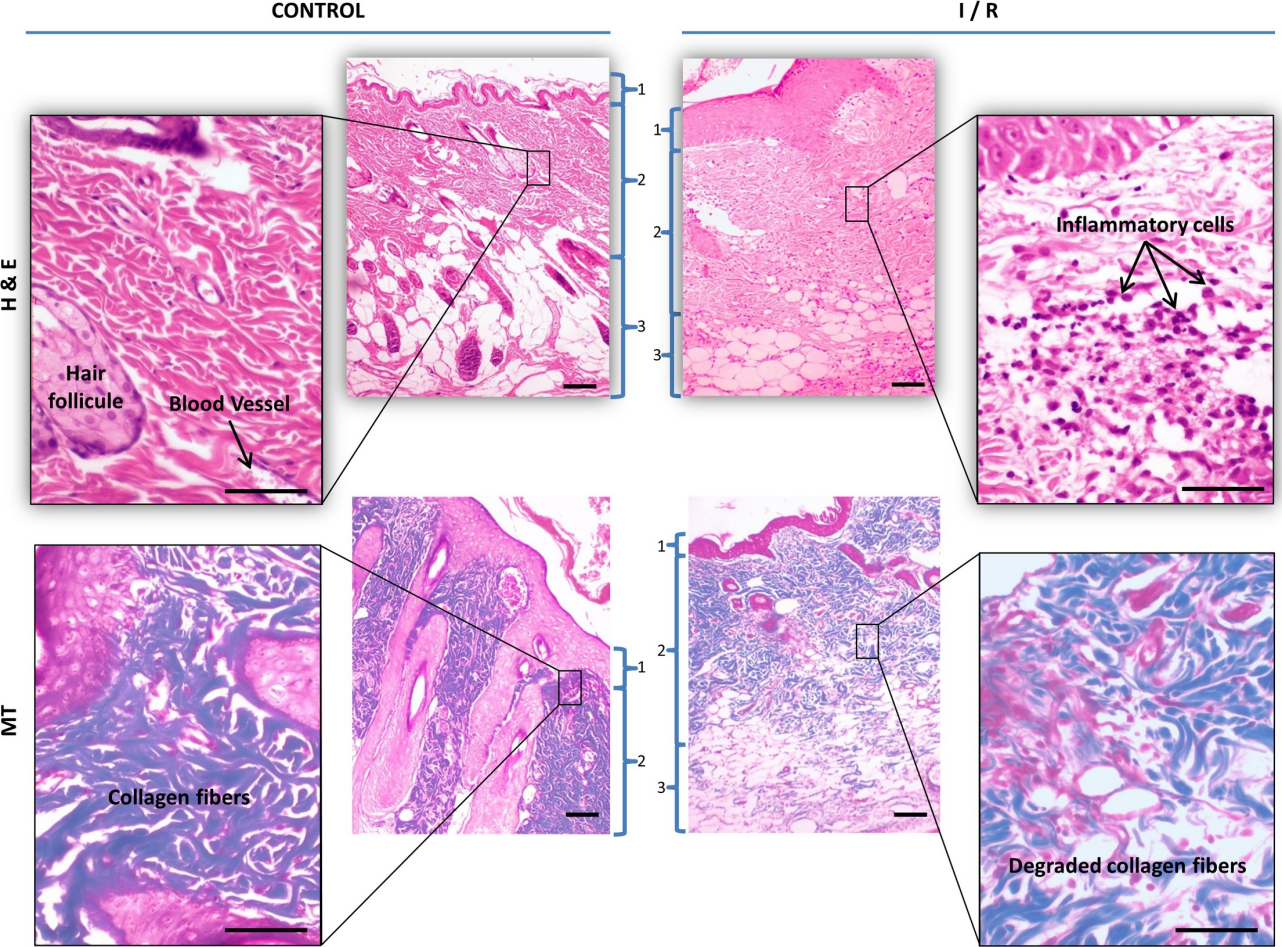
Representative histological images. At day 7 post-surgery, the skin flaps of euthanized animals were fixed in 4% paraformaldehyde, paraffin-embedded, and stained for hematoxylin-eosin (H&E) and Masson's trichrome (MT). Horizontal bars represent 100 μm. The different skin layers are numbered: epidermis (1), dermis (2) and adipose tissue (3).

The histology of the distal flap area (Q1 quadrant from Fig 3) exhibited full-thickness skin necrosis, structural damage, subcutaneous edema and a large number of infiltrated inflammatory cells in all those flaps that suffered 8 hours of ischemia. In contrast, control skin flaps showed much less inflammatory cell infiltration (Fig 5).

The histology scores showed that the level of infiltration of neutrophils in the control group animals was significantly lower than that in the I/R group (p < 0.05). Also, a significant increase in necrosis severity was found in the I/R group when compared with that in the control group (p < 0.05) (Table 2).

**Table 2:** Histological scores in control and I/R groups. Differences were statistically analyzed using Student’s t-test for variables with a parametric distribution and Mann-Whitney’s U-test for non-parametric variables. *p-values <0.05 were considered to indicate statistical significance.

		Control	I/R
**Inflammatory-related cells**	Neutrophils	1.571 ±0.787	2.714 ±0.488 *
	Eosinophils	1.000 ±0.000	1.000 ±0.000
	Lymphocytes	1.429 ±0.535	1.143 ±0.378
	Plasma cells	1.000 ±0.000	1.000 ±0.000
	Macrophages	2.000 ±0.000	1.429 ±0.535
	Mastocytes	1.143 ±0.378	1.000 ±0.000
	Giant cells	1.286 ±0.488	1.143 ±0.378
**Tissue damage parameters**	Necrosis	0.143 ±0.378	2.286 ±1.254 *
	Edema	1.143 ±0.690	1.429 ±0.787
	Hemorrhage	0.714 ±0.951	1.429 ±1.397
	Thrombosis	0.286 ±0.488	0.857 ±0.900
**Collagen deposition**	Fibrosis	1.571 ±0.535	1.714 ±0.756
	Vascular proliferation	2.714 ±0.488	2.143 ±0.900

### Gene expression analysis

**Fig 6:**
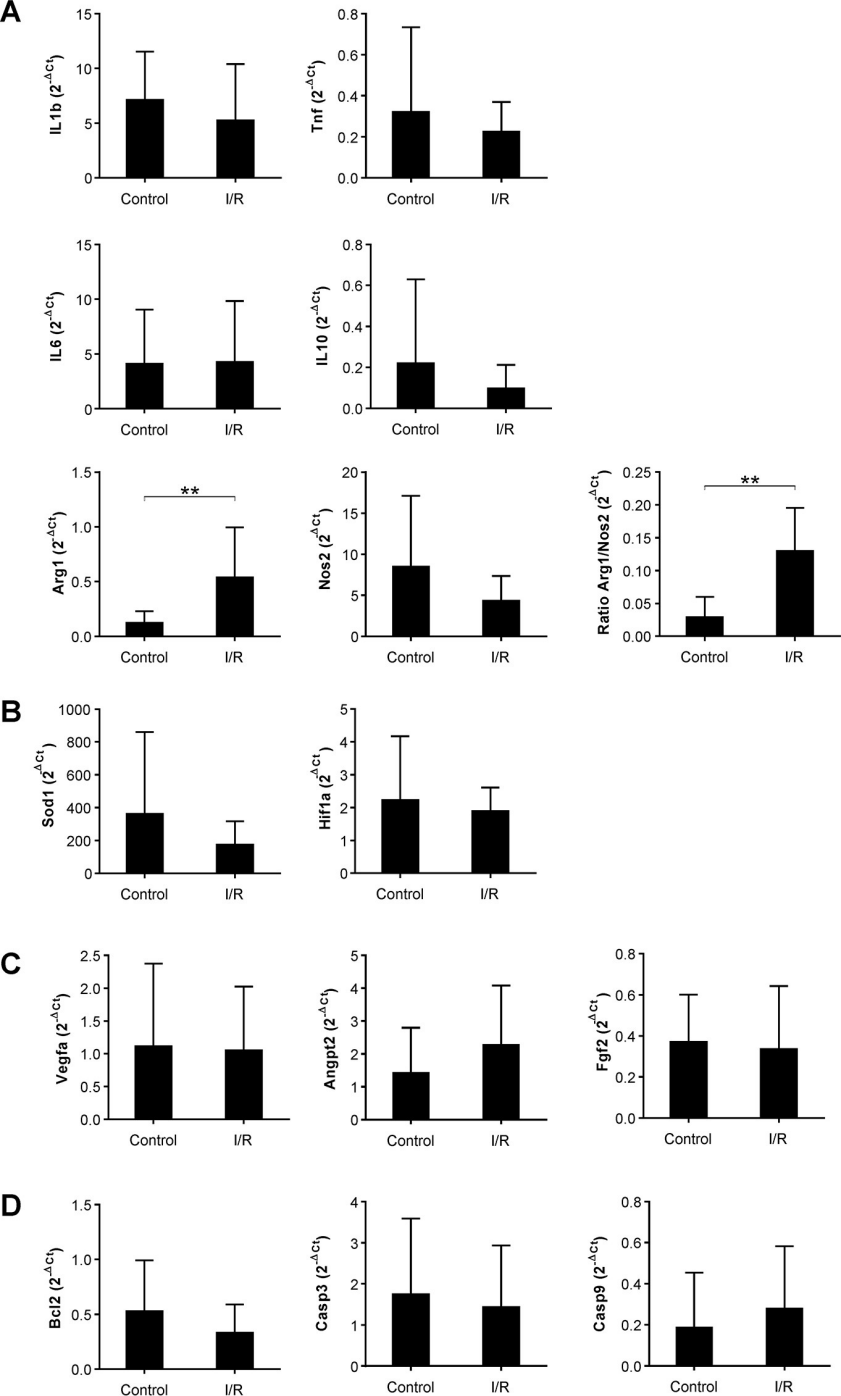
Quantitative expression of ischemia-reperfusion-related genes. At day 7 post-surgery, total RNA from the skin flaps was isolated and qRT-PCR products were quantified by the 2^-ΔCt^ method. Graphs represent the mean ± SD of independently performed experiments. The results were organized by groups of genes related with the following biological processes: A) Inflammatory response, B) Oxidative stress, C) Angiogenesis, and D) Necrosis/Apoptosis. Data were statistically analyzed using Student’s t-test for variables with a parametric distribution and Mann-Whitney’s U Test for non-parametric variables. Horizontal bars represent statistically significant differences (**p<0.01).

Expression of genes related with inflammatory response, angiogenesis, necrosis/apoptosis and oxidative stress was quantified in the harvested tissues (Q4 quadrant from Fig 3). No significant differences were found between groups when we compared expression of the following inflammation-related genes: IL1b, IL6, IL10, Tnf and iNOS. Significant difference was found for angiogenesis-related genes: angiopoietin-2, Fgf2 and Vegfa. Similarly, expression of oxidative stress-related genes (SOD1, HiF1a) and apoptosis/necrosis-related genes (Cas3, Cas9, Bcl2) did not show significant differences. Interestingly, our results demonstrated that Arginase 1 as well as the Arginase 1/Nitric Oxide Synthase 2 ratio showed a significant increase in expression in the I/R group (p<0.01) (Fig 6).

## Discussion

Ischemia is inevitable in all microsurgical free flaps performed in reconstructive procedures [40]. The ischemia period is closely related with an inflammatory response, which is accelerated and augmented when the ischemic tissue is reperfused [4]. The process is dependent on a complex interaction between a variety of inflammatory mediators [51].

In this work, we have tried to improve the characterization of an animal model to study ischemia-reperfusion injury in the field of reconstructive surgery. The use of our refined preclinical model will hopefully instill greater confidence in clinicians when deciding which compounds are worthy of further investigation in preclinical or human clinical trials. The characterization of this microsurgical model for preclinical research is based on five different aspects: anatomy, ischemia-reperfusion injury induction, blood flow assessment, histology and gene expression.

In our ischemia/reperfusion injury animal model, after the anatomical dissections, vascular ligatures and anastomoses, hindlimb blood flow was not compromised in any animal. Furthermore, we did not observe any resulting pain or limp. In agreement with *Kochi et al* [48], our research model left intact three collateral routes through intramuscular networks in the quadriceps femoris muscle, biceps femoris muscle and the medial thigh muscles including the medial hamstring muscles and adductor muscles.

In our study, the surgical procedure was performed by microsurgical anastomoses to induce I/R injury. Although it is a time-consuming procedure that requires fine microsurgical skills, flaps undergoing anastomoses are more resistant to ischemia than those undergoing clamping [52]. Moreover, our surgical methodology is more translatable to a clinical scenario in free flap surgery.

During the surgical procedures, we have measured the blood flow using transit-time ultrasound technology, which is a novel and objective method of evaluation of microsurgical anastomoses [53,54]. Blood flow measurement is of crucial importance in microsurgery [55], as part of the tissue damage due to ischemia is responsible for slowing down blood flow and causing thrombosis [56]. The widely used manual patency test demonstrates whether there is blood flow through anastomoses sites, but it does not demonstrate the quality of the blood flow [57]. Transit-time flow measurement allows a quantitative and objective assessment of vascular function and helps to prevent failure of anastomoses [54,58,59]. Our research model ensured in all animals a good microvascular perfusion after 8 hours of ischemia (no statistical differences were observed). This test objectively demonstrated that our macroscopic, histological and genetic results were neither influenced by blood perfusion differences nor by the microsurgical technique, which is is essential to delineate valuable conclusions from our ischemia/reperfusion study.

The anatomopathological analysis of the free flaps at day 7 revealed infiltration of leukocytes, structural damage and edema in all those flaps that suffered 8 hours of ischemia. Control skin flaps had lower inflammatory cell infiltration than in the animals of I/R group (p < 0.05). Furthermore, a significant increase in necrosis severity was found in I/R group when compared with that in the control group (p < 0.05). These results are in agreement with those of previous studies in the area of ischemia/reperfusion injury [20,47,60].

Once we identified the histological changes, we next aimed to characterize the gene expression pattern of tissue samples from the control and I/R groups. The main goals of this analysis were to obtain an overview of the pathophysiology of ischemia reperfusion injury and to identify molecular biomarkers for preclinical studies and drug testing.

Interestingly, our analysis revealed the absence of significant changes in Th1/Th2 cytokine levels, which may indicate that most probably that other Th1/Th2 cytokines (i.e., IFN-γ, IL-2 or IL-4) may have differences but they were initially excluded from this study. Regarding to M1/M2–related mRNAs, the expression of Arginase 1 as well as the ratio Arginase 1/Nitric Oxide Synthase 2 showed a significant increase in the I/R group. Arginase 1 is released by M2 macrophages to metabolize L-arginine into ornithine and urea, and its expression is widely used as a classic M2 marker [61]. The significant increase of Arginase 1 expression may reflect a macrophage polarization towards and anti-inflammatory phenotype, however, it is interesting to note that the anatomopathological analysis demonstrated a decrease in the number of infiltrated macrophages in the I/R group. Considering these two changes (decrease in the number of infiltrating macrophages and increase of Arginase-1 expression), here we hypothesize that, in our experimental model, the I/R injury triggered the migration of M1 macrophages towards apoptotic and necrotic areas. Simultaneously, the viable tissue counteracted the necrotic response retaining pro-regenerative M2 cells to promote tissue regeneration. Additionally, the analysis of Arginase-1 expression in this I/R model could also be considered a useful biomarker for proof of concept studies and for the development of efficient therapies, but this aspect will be further discussed.

Apart from inflammatory-related genes, this study was also focused on three biological process associated with I/R injury: angiogenesis, oxidative stress and apoptosis. As shown in the results section, any significant difference was observed when these genes were compared between groups. The absence of significant differences among groups could reflect the maintenance of tissue homeostasis during ischemic insult in this I/R model. However, taking into account our macroscopic and histopathological results in this animal model, we should also consider the possibility that there are myriad genes that may have a key role in angiogenesis, oxidative stress or apoptosis and were not included in the selected panel of genes in this study.

Finally, it is important to highlight the relevance of the differential expression of Arginase 1 in the I/R free flap model. It is well known that the induction and activation of arginase is a common event that occurs in the initial stage of ischemic events [62,63].

The hypoxia and ischemic conditions shift arginine to be metabolized to ornithine and urea. This shift has been previously described in ischemic heart disease [63], renal ischemia-reperfusion injury [64] as well as in neurovascular injury after retinal I/R [65].

Given that arginase and iNOS is tightly regulated by direct protein modification or by induction of enzyme expression, our study was also focused on the simultaneous quantification of iNOS and their ratio.

## Conclusion

Our results demonstrated an imbalance of arginase/iNOS ratio (with a predominant arginase activity), that could be interpreted as a biomarker to identify the initial phase of I/R injury in the tissue. It is important to note the limitations of this study lies in the fact that there are numerous genes that were not included in the qPCR analysis. In this sense, we should not exclude the importance of other biomarkers such as miRNAs or siRNAs with key role in angiogenesis, oxidative stress or apoptosis.

Additionally, it is important to point out the importance of this animal model to evaluate different therapies to counteract ischemia-reperfusion injury in skin free flaps. In particular, considering that arginase inhibition has been hypothesized as a promising therapeutic strategy for the treatment of I/R injury [66,67], this animal model could be especially useful for preclinical studies focused on the evaluation of arginase inhibitors such as NO scavengers or NOS inhibitors for a clinically safe ischemia time in free flap surgery.
